# Case series on clinical applications of liquid biopsy in pediatric solid tumors: towards improved diagnostics and disease monitoring

**DOI:** 10.3389/fonc.2023.1209150

**Published:** 2023-08-17

**Authors:** Nina U. Gelineau, Astrid van Barneveld, Atia Samim, Lieke Van Zogchel, Nathalie Lak, Michelle L. Tas, Yvette Matser, Annelies M. C. Mavinkurve-Groothuis, Martine van Grotel, Jószef Zsiros, Natasha K. A. van Eijkelenburg, Rutger R. G. Knops, Roelof van Ewijk, Karin P. S. Langenberg, Ronald De Krijger, Laura S. Hiemcke-Jiwa, Ruben Van Paemel, Lotte Cornelli, Katleen De Preter, Bram De Wilde, Ellen Van Der Schoot, Godelieve Tytgat

**Affiliations:** ^1^ Princess Máxima Center for Pediatric Oncology Research, Utrecht, Netherlands; ^2^ Department of Experimental Immunohematology, Sanquin Research, Amsterdam, Netherlands; ^3^ Department of Pathology, University Medical Center Utrecht, Utrecht, Netherlands; ^4^ Department of Biomolecular Medicine, Ghent University, Ghent, Belgium; ^5^ Department of Pediatric Hematology, Oncology and Stem Cell Transplantation, Ghent University, Ghent, Belgium; ^6^ Research Institute, Ghent University, Ghent, East Flanders, Belgium; ^7^ VIB-UGent Center for Medical Biotechnology, Gent, Belgium

**Keywords:** liquid biopsy, pediatric cancer, qPCR, ddPCR, RRBS, solid tumor, mRNA, cfDNA (cell-free DNA)

## Abstract

**Background and aims:**

Solid tumors account for about 30% of all pediatric cancers. The diagnosis is typically based on histological and molecular analysis of a primary tumor biopsy. Liquid biopsies carry several advantages over conventional tissue biopsy. However, their use for genomic analysis and response monitoring of pediatric solid tumors is still in experimental stages and mostly performed retrospectively without direct impact on patient management. In this case series we discuss six clinical cases of children with a solid tumor for whom a liquid biopsy assay was performed and demonstrate the potential of liquid biopsy for future clinical decision making.

**Methods:**

We performed quantitative real-time PCR (RT-qPCR), droplet digital PCR (ddPCR) or reduced representation bisulphite sequencing of cell-free DNA (cfRRBS) on liquid biopsies collected from six pediatric patients with a solid tumor treated between 2017 and 2023 at the Princess Máxima Center for Pediatric Oncology in the Netherlands. Results were used to aid in clinical decision making by contribution to establish a diagnosis, by prognostication and response to therapy monitoring.

**Results:**

In three patients cfRRBS helped to establish the diagnosis of a rhabdomyosarcoma, an Ewing sarcoma and a neuroblastoma (case 1-3). In two patients, liquid biopsies were used for prognostication, by MYCN ddPCR in a patient with neuroblastoma and by RT-qPCR testing rhabdomyosarcoma-specific mRNA in bone marrow of a patient with a rhabdomyosarcoma (case 4 and 5). In case 6, mRNA testing demonstrated disease progression and assisted clinical decision making.

**Conclusion:**

This case series illustrates the value of liquid biopsy. We further demonstrate and recommend the use of liquid biopsies to be used in conjunction with conventional methods for the determination of metastatic status, prognostication and monitoring of treatment response in patients with pediatric solid tumors.

## Introduction

Solid tumors account for about 30% of all pediatric cancers. The most common extra-cranial solid tumors in children are neuroblastoma, rhabdomyosarcoma, Wilms tumor and bone sarcomas (1). In general, solid tumors are diagnosed by histological and molecular profiling of the primary tumor biopsy. However, tissue analysis provides only a snapshot of the tumor, overlooking any spatial and temporal heterogeneity within the tumor or between the primary lesion and metastases (2–12). Disease monitoring and follow-up is generally performed with imaging, supplemented with tumor-specific biomarkers in a small number of tumor types (13–15). However, anatomical and functional (nuclear) imaging has limited sensitivity and is only capable of detecting lesions of at least 0.5-1 cm, with 1 cm (3) of solid tissue containing ~10 (9) cells (16), highlighting the need for more sensitive techniques to detect minimal residual disease (MRD).

Liquid biopsies allow detailed analysis of the tumor profile in body fluids. Circulating sources of genomic information, such as cell-free tumor DNA (ctDNA), circulating tumor cells and tumor-derived mRNA, can reveal characteristics that are relevant for clinical decision making, but can be absent in a tumor biopsy (17–19). Liquid biopsies are often minimally invasive, which enables repeated sampling to monitor response to therapy, tumor load and clonal evolution over time. In addition, most liquid biopsies do not require general anesthesia, are less dependent on cancer stage and localization and are cheaper than tissue biopsies (20).

**Figure 1:**
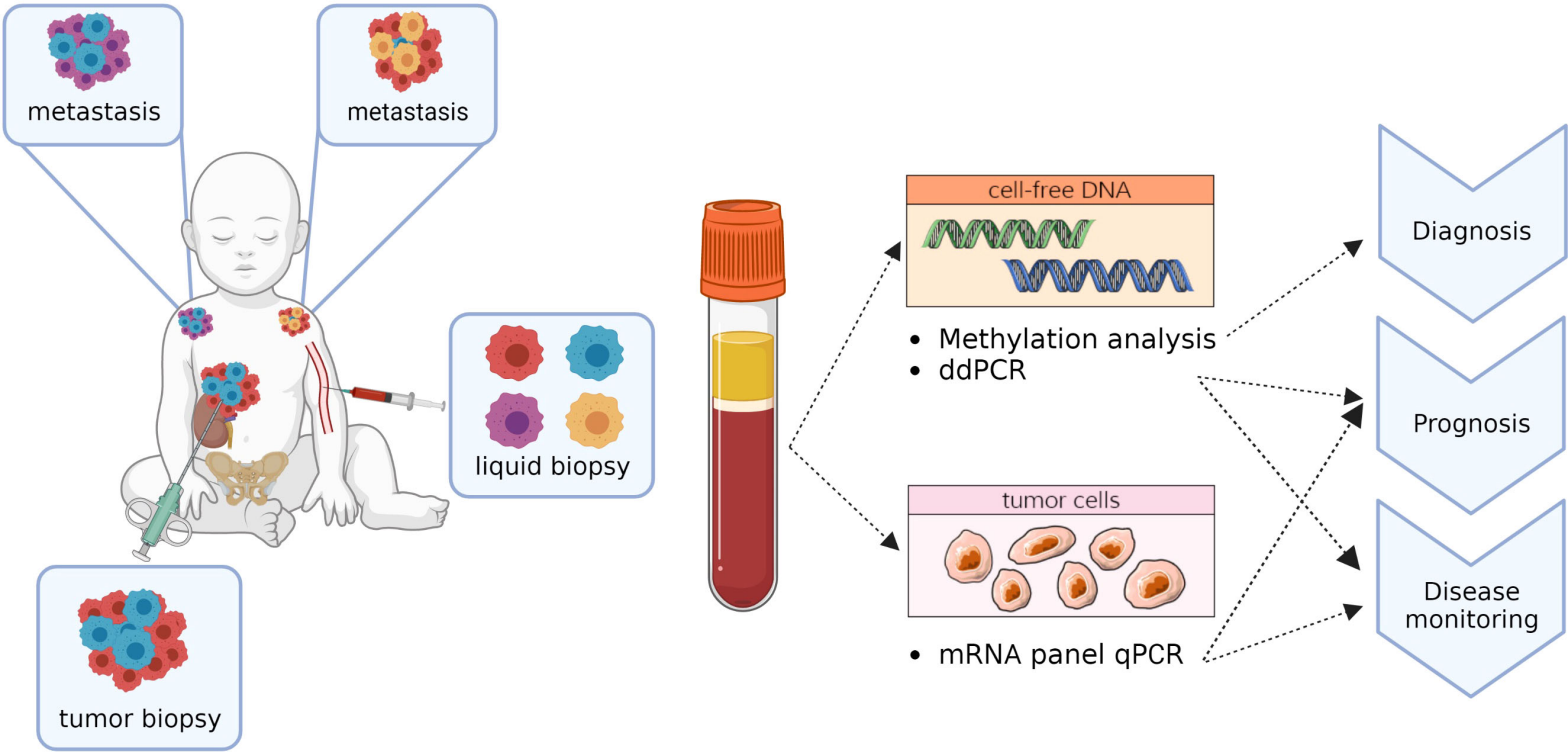
Potential applications of liquid biopsy components.

Currently, the use of liquid biopsies for genomic analysis and monitoring of pediatric solid tumors is still in experimental stages and mostly performed retrospectively without direct impact on patient management. However, incidental cases do demonstrate their potential for future clinical decision making. Many techniques that are classically used for tissue analysis can also be applied to liquid biopsies, including various sequencing approaches for cell-free DNA (cfDNA), tumor-specific PCR (both quantitative real-time PCR (RT-qPCR) and digital PCRs) and methylation analysis (**Figure 1**) (21–24).

In this report, we describe the clinical application of RT-qPCR, droplet digital PCR (ddPCR) and methylation analysis through reduced representation bisulphite sequencing of cell-free DNA (cfRRBS). RT-qPCR has been shown to be a sensitive technique for the detection and monitoring of MRD in several pediatric solid tumors (25–27). Our group previously demonstrated that tumor-specific mRNA panels for neuroblastoma sensitively detect neuroblastoma-specific mRNAs in bone marrow (BM) and peripheral blood (PB) that correlate with outcome (28, 29). Similar observations were made in rhabdomyosarcoma, where RT-qPCR positivity for tumor-specific mRNA in BM at diagnosis correlated with poor prognosis for both metastatic and non-metastatic patients (30).

ddPCR is a digital PCR method with high sensitivity and specificity that produces reliable results even with low input amounts of cfDNA (31, 32). In neuroblastoma, where amplification of *MYCN* is an indicator for high-risk disease (33), *MYCN* copy number status can be determined by ddPCR using cfDNA in blood plasma. In addition, ddPCR can be used to detect specific epigenetic abnormalities such as methylated *RASSF1A* (*RASSF1A*-M). *RASSF1A* is the most frequently inactivated tumor suppressor gene in cancer, most often inactivated by hypermethylation of the promotor (34, 35). At diagnosis, *RASSF1A*-M levels correlate with poor outcome in neuroblastoma and rhabdomyosarcoma. The marker also shows potential for sensitive MRD detection in neuroblastoma (32, 36).

Finally, cfRRBS is a promising technique recently developed at Ghent University, Belgium, that capitalizes on the specificity of tissue methylation patterns. Like healthy tissue, cells of the same tumor type share a similar, detectable methylation pattern, although differences do exist between subtypes (37, 38). In their proof-of-concept study, Van Paemel et al. were able to accurately determine a diagnosis for multiple pediatric tumor types by analyzing their methylation patterns using cfRRBS (39).

Here, we discuss six clinical cases of children with a solid tumor for whom a liquid biopsy assay was performed and provide a summary of existing literature on the techniques presented in this paper, with a particular focus on their use in pediatric solid tumors.

## Methods

### Patients and samples

All patients underwent treatment at the Princess Máxima Center for Pediatric Oncology between 2017 and 2023. Samples from patients were either prospectively obtained or retrospectively collected from biobanked material if informed consent from parents or guardians was obtained according to the declaration of Helsinki. BM-aspirates were collected from bilateral sites in EDTA tubes and processed within 24 hours after collection and stored in Trizol (Invitrogen, Carlsbad, CA) at -80°CC. PB samples were collected and processed the same way and stored in in PAXgene blood RNA tubes (QIAGEN, Venlo, the Netherlands) at -20°CC, or in Trizol (Invitrogen, Carlsbad, CA) at -80°CC. DNA and RNA isolation and cDNA synthesis was performed as previously described (30, 32, 40).

### Quantitative real-time PCR

For the patients included in this report, tumor-derived mRNA was detected in the cellular fraction of bone marrow (BM) aspirates and/or peripheral blood (PB). For neuroblastoma, a multiplex (MPX) adrenergic marker panel was used which includes *PHOX2B*, *TH* and *CHRNA3*, and *GAP43* and *DBH* for BM and PB (28, 29). For rhabdomyosarcoma, the MPX panel used for BM includes *MYOD1*, *MYOG*, *PDLIM3*, *ACTC1*, *ZIC1* and *PAX3/7-FOXO1* and for PB, *SNAI2*, *CDH11*, *THEM47* and *MEGF10* (30). Expression of the markers was normalized to *GUSB* expression and positivity was scored according to thresholds published by Van Zogchel et al. and Lak et al. (28–30) To calculate the level of infiltration in the neuroblastoma samples, expression levels of the mRNA markers in BM or PB were related to the expression in neuroblastoma cell line IMR32.

### Droplet digital PCR

Detection of *RASSF1A*-M in ctDNA was performed using the method described by Van Zogchel et al. (32, 41) This method subjects every sample to two ddPCR reactions after cfDNA extraction, once with methylation-sensitive restriction enzymes (MRSE) added and once without. This allows for accurate discrimination between methylated and unmethylated *RASSF1A*. As an input control, two assays for the reference gene *ACTB* are used of which one amplicon is digested by the enzymes and one is not.

For the patients in this report, a *MYCN* primer-probe set and an *NAGK* (normal diploid reference gene) primer-probe set were added in a duplex PCR reaction. *MYCN* copy number was then determined by calculating the ratio of *MYCN* to the reference gene (42, 43).

### Reduced representation bisulphite sequencing of cell-free DNA

Methylation analysis of cfDNA using cell-free reduced representation bisulphite sequencing (cfRRBS) was performed as previously described (39). The diagnosis is established based on the highest estimated tumor fraction using reference-based deconvolution as described by Moss et al. (39).

See **Supplementals** for a detailed description of patients and methods.

## Case descriptions

For a summary of the conventional diagnostics and clinical value of liquid biopsy for each case, see **Table 1**.

**Table 1:** The added value of liquid biopsy in these cases.

	Conventional diagnostics	Research diagnostics
Diagnosis		
**Case 1** 3-year old female; renal tumor with lung and suspected BM metastases on imaging. Pathology: Regressive type Wilms tumor. Progression with leptomeningeal lesions of unknown origin.	Cerebral MRI: multifocal perivascular and leptomeningeal enhancement	Initial diagnostic plasma sample (1 ml): cfDNA ➔ methylation by cfRRBS: embryonal rhabdomyosarcoma (81%)
	Cerebral CT: midline shift and cerebrum and leptomeningeal enhancement, suspicious for metastases	
	Cerebrospinal fluid: atypical cells of unclear origin	
	Tumor biopsy: not performed due to procedural difficulty, performed postmortem	
	Tumor biopsy postmortem: - Immunohistochemical analysis: small blue round cell tumor negative for Keratin7, PAX8 and WT1; Myogenin and MyoD1 positive - Methylation analysis (EPIC array): embryonal rhabdomyosarcoma (calibration score 0.99)	
**Case 2** 11-year old male; Differential diagnosis included neuroblastoma and rhabdoid tumor	Urinary catecholamines: HVA and VMA not elevated; dopamine, 3MT and norepinephrine elevated	Initial diagnostic plasma sample (1 ml): cfDNA ➔ methylation by cfRRBS: rhabdoid tumor (44.2%), neuroblastoma (22.8%)
	Abdominal MRI: retroperitoneal mass in the right adrenal gland suspect for rhabdoid tumor, neuroblastoma, or soft-tissue sarcoma	Initial diagnostic BM and PB sample (500 µL): mRNA → PB and BM RT-qPCR panels: neuroblastoma markers positive
	[^123^I]mIBG: heterogeneous MIBG uptake	
	Tumor biopsy: - Immunohistochemical analysis: PHOX2B and TH negative, Chromogranin A negative - SNP-array: gain chromosome 17q - Next-generation sequencing: *SMARCA4* mutation	
	DNA methylation analysis (Institutional classifier of Heidelberg): rhabdoid tumor (score 0.73)	
	Trephines: no BM invasion	
	Tumor resection post induction chemotherapy: focal ganglion cells, conclusive for neuroblastoma	
**Case 3** 2-year old male; Large thoracic mass, no tumor biopsy possible at diagnosis	Thoracic CT: lytic lesion 7^th^ rib, multiple masses in pleura and vertebrae and a filled right hemithorax with mediastinal shift and deviation of the trachea and left main bronchus	Initial diagnostic plasma sample (1 ml): cfDNA ➔ methylation analysis by cfRRBS: Ewing sarcoma (28%)
	Tumor biopsy (at diagnosis): not feasible due to respiratory risk	
	Tumor biopsy (after chemotherapy): Ewing sarcoma	
Prognosis		
**Case 4** 6-week old female; suspicion of neuroblastoma with enlarged liver. Small primary tumor, no tumor biopsy possible.	Urinary catecholamines: VMA, HVA, dopamine, 3MT, norepinephrine and normetanephrine levels elevated	Initial diagnostic BM and PB sample (500 µL): mRNA ➔ RT-qPCR panels: all neuroblastoma markers positive
	Abdominal MRI: small tumor in the right adrenal gland with massive liver infiltration	Initial diagnostic plasma sample (1 ml): cfDNA ➔ ddPCR amplification-assay: no indication of MYCN amplification
	[^123^I]mIBG: not performed at diagnosis due to clinical condition necessitating immediate start of chemotherapy	
	Tumor biopsy: not performed due to risk of respiratory and circulatory failure	
**Case 5** 5-year old female; rhabdomyosarcoma left arm, BM infiltration could not be determined	[^18^F]FDG-PET: tumor of the soft tissue in left forearm without metastases	Initial diagnostic BM sample (500 µL): mRNA ➔ RT-qPCR panel: all rhabdomyosarcoma markers negative
	Tumor biopsy: embryonal rhabdomyosarcoma	
	Bone marrow aspirates: MyoD1 staining ambiguous	
Disease monitoring		
**Case 6** 6-year old male with recurrent neuroblastoma, clinical suspicion of tumor progression post MIBG therapy	Urinary catecholamines: not analyzed due to bladder irrigation	PB sample at event (500 µL): mRNA ➔ RT-qPCR panel: all neuroblastoma markers positive
	[^123^I]mIBG: not performed post [^131^I]mIBG therapy	Plasma sample at event (1 mL): cfDNA ➔ ddPCR hypermethylation-assay: *RASSF1A* hypermethylation positive
	[^18^F]FDG-PET: no differentiation possible between progressive disease with BM infiltration and BM activity secondary to radionuclide therapy	

### Case 1

**Figure 2:**
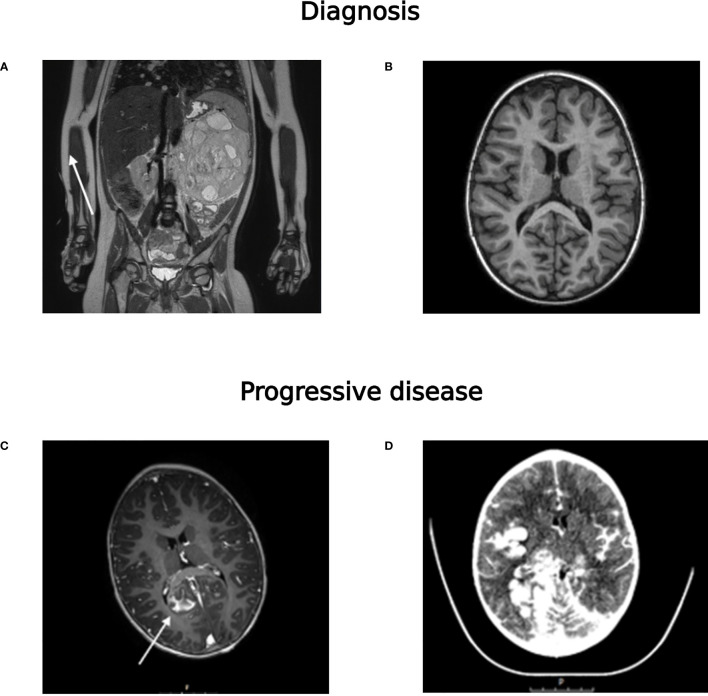
**(A)** MRI-scan (T2-TSE) at diagnosis showing the abdominal tumor, pulmonary metastases (indicated with an arrow) and a metastasis in the right proximal femur (indicated with an arrow), **(B)** MRI-scan of the brain (T1) at diagnosis, **(C)** after start of neurological symptoms (T1-gadolinium), showing extensive leptomeningeal metastases (indicated with an arrow) and **(D)** CT-scan (with ultravist contrast) shortly before death of the patient.

A 3-year-old girl presented with lethargy, abdominal pain, vomiting and a palpable abdominal mass (**Table 1**). An MRI-scan revealed a tumor of the left kidney with several pulmonary metastases and skeletal lesions, suspicious for metastases (**Figure 2A**). A fine needle tissue biopsy performed to differentiate between clear cell sarcoma (CCSK) and Wilms tumor showed extensive necrosis and was of no diagnostic value. An MRI of the brain showed no abnormalities (**Figure 2B**) and bone marrow testing showed no metastases. Chemotherapy was started empirically according to the SIOP-RTSG UMBRELLA trial CCSK regimen. At nephrectomy, the tumor was identified as a metastatic regressive type-intermediate risk Wilms tumor. During post-operative chemotherapy, the patient developed progressive bradycardia, vomiting, eye movement disorders and epileptic seizures. Imaging (by MRI and CT-scan) of the brain showed new leptomeningeal lesions (**Figures 2C, D**) that could not be biopsied. Analysis of cerebrospinal fluid showed atypical cells of unclear origin. Again, a definitive diagnosis could not be made. Shortly thereafter, the patient died. Postmortem analysis of the intracerebral lesions showed a small blue round cell tumor which was negative for Wilms tumor markers Keratin7, PAX8 and WT1. Methylation analysis (cfRRBS) of cfDNA isolated from the bio-banked plasma sample taken at diagnosis estimated a second primary tumor, an embryonal rhabdomyosarcoma (81% estimated tumor fraction). This was later confirmed by methylation analysis by EPIC array and immunohistochemical analysis of the postmortem biopsy. Prompted by the simultaneous occurrence of two primary tumors, whole-exome sequencing (WES) was performed for additional germline analysis. No aberrations were detected.

### Case 2

**Figure 3:**
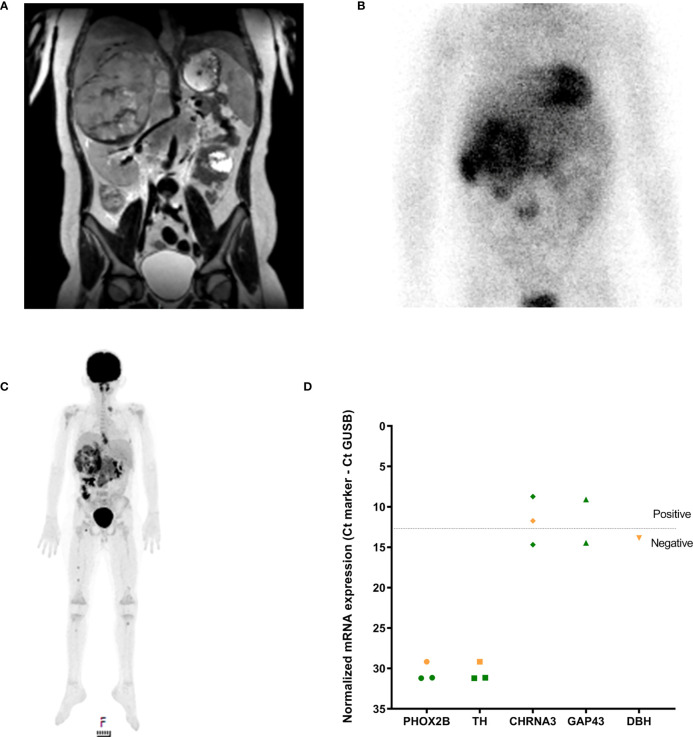
**(A)** MRI-scan (T2-TSE) at diagnosis showing the abdominal tumor, **(B)** [^123^I]mIBG-scan and **(C)** FDG-PET at diagnosis and **(D)** RT-qPCR neuroblastoma mRNA marker-panel results at diagnosis (Y-axis, normalized mRNA expression noted as Ct_marker_ - Ct_GUSB_; green BM infiltration, orange PB infiltration). Dashed line indicates separation of positivity.

An 11-year-old boy presented with fever, vomiting, abdominal pain and severe back pain (**Table 1**). MRI (**Figure 3A**) showed a retroperitoneal tumor extending into the right suprarenal area, with locoregional lymph node involvement and a potential skeletal metastasis in the S1 vertebra. The differential diagnosis included rhabdoid tumor, neuroblastoma and soft-tissue sarcoma. On [^123^I]mIBG imaging (**Figure 3B**) there was heterogeneous [^123^I]mIBG uptake of the primary tumor, with large [^123^I]mIBG negative parts of the primary tumor and locoregional lymph node metastases. In contrast to the MRI, multiple skeletal metastases with faint [^123^I]mIBG uptake (SIOPEN score of 4) were detected. Because of the heterogeneous [^123^I]mIBG uptake, an [^18^F]FDG-PET CT was performed (**Figure 3C**), confirming all lesions on [^123^I]mIBG imaging plus identifying additional skeletal lesions, as suspected on the MRI. Analysis of the urinary catecholamines showed normal homovanillic acid (HVA) and vanillylmandelic acid (VMA) and slightly elevated levels of dopamine, 3-methoxytyramine (3MT) and norepinephrine. Histologically, the primary tumor biopsy did not express common neuroblastoma markers (negative for PHOX2B and TH, but positive for Chromogranin A). SNP-array showed a gain of chromosome 17q and next-generation sequencing detected a *SMARCA4* mutation [c.4574T>C; p.(Leu1525Pro)]. Based on DNA-methylation (using the institutional classifier of Heidelberg), the tumor was classified as a rhabdoid tumor (score 0.73). Methylation analysis of cfDNA from the blood plasma was performed to aid in the diagnosis of this case. Deconvolution of cfRRBS data estimated both rhabdoid tumor (44.2% tumor fraction) and neuroblastoma (22.8% tumor fraction). In addition, RT-qPCR was performed on diagnostic BM and PB samples using the neuroblastoma MPX-panel, which came back positive for mRNA markers *CHRNA3* and *GAP43* (**Figure 3D**). At tumor resection following induction chemotherapy, tumor cells stained positive for chromogranin A and synaptophysin and focal ganglion cells, suggestive of neuroblastoma. The diagnosis was amended and the patient received postoperative chemotherapy according to the GPOH NBL2009 high-risk neuroblastoma regimen.

### Case 3

**Figure 4:**
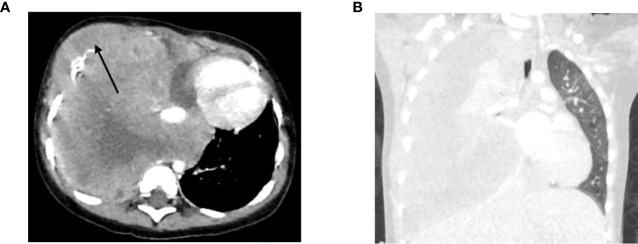
**(A)** Axial CT-scan (soft tissue setting) at diagnosis showing the primary tumor (indicated with an arrow) and **(B)** coronal CT-scan (lung setting) at diagnosis showing the pleural effusion.

A 2-year-old boy presented with progressive episodes of coughing, dyspnea and a palpable mass on the chest (**Table 1**). A CT-scan showed multiple masses of the rib, vertebrae and pleura, and a filled right hemithorax (pleural effusion) with a mediastinal shift and deviation of the trachea and left main bronchus (**Figure 4**). The subsequent risk of respiratory failure during anesthesia precluded tumor biopsy at diagnosis. Methylation analysis using cfRRBS on the cfDNA at diagnosis estimated a fraction of Ewing sarcoma (28% tumor fraction). After the first chemotherapy course the tumor responded and a biopsy was feasible, and the diagnosis of an Ewing sarcoma was confirmed.

### Case 4

**Figure 5:**
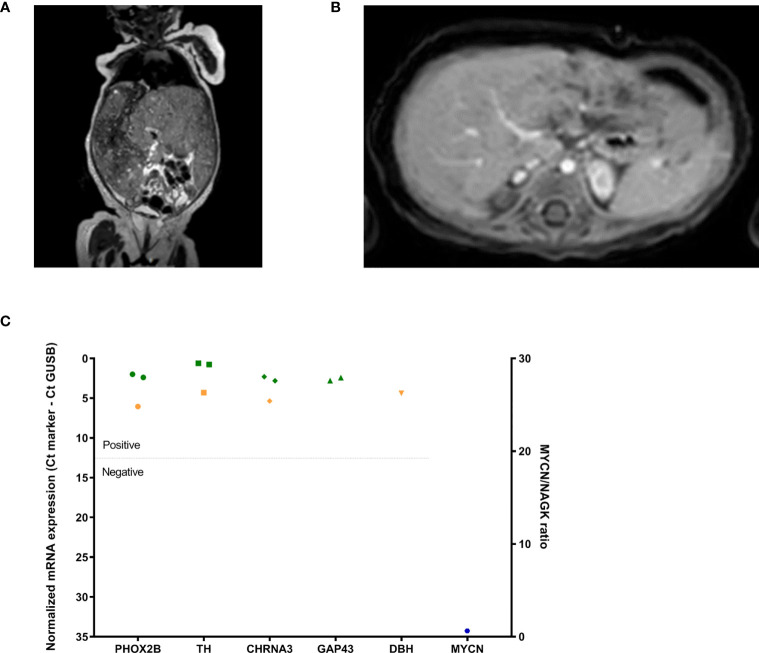
**(A)** MRI-scan (T2 weighted) at diagnosis showing the abdominal and enlarged liver, **(B)** after treatment (MRI after gadolinium injection) showing gadolinium enhancement of the minimal rest tumor (indicated with an arrow and circle) and **(C)** RT-qPCR neuroblastoma mRNA marker-panel results at diagnosis (left Y-axis, normalized mRNA expression noted as Ct_marker_ - Ct_GUSB_; green BM infiltration, orange PB infiltration) and ddPCR *MYCN* amplification-assay (right Y-axis, ratio *MYCN*/*NAGK*, blue hexagon).

A 6-week-old girl presented with abdominal distention and a palpable abdominal mass (**Table 1**). MRI showed a small tumor in the right adrenal gland with massive liver infiltration, resulting in a very large liver that extended into the pelvis (**Figure 5A**). The progressive hepatomegaly caused feeding problems, respiratory distress and edema of the lower abdomen and legs. Analysis of urinary catecholamines showed elevated VMA, HVA, dopamine, 3MT, norepinephrine and normetanephrine levels. As the patient’s clinical condition did not allow for an [^123^I]mIBG SPECT scan or tumor biopsy to be performed, chemotherapy was immediately started. After chemotherapy, only a very small (8x10 mm) calcified primary tumor lesion remained (**Figure 5B**). RT-qPCR of BM and PB samples at diagnosis showed detection of all neuroblastoma mRNA-markers, including the neuroblastoma-specific marker *PHOX2B* (**Figure 5C**). To be able to discriminate between high- and non-high-risk disease, a ddPCR to detect *MYCN* status was performed on cfDNA isolated from plasma at diagnosis. No amplification was detected (**Figure 5C**), and a non-high risk neuroblastoma was diagnosed. The patient was treated with chemotherapy according to a medium-risk protocol. The patient responded very well to treatment and is still in complete remission, indicating that the clinical course is in line with the risk stratification.

### Case 5

**Figure 6:**
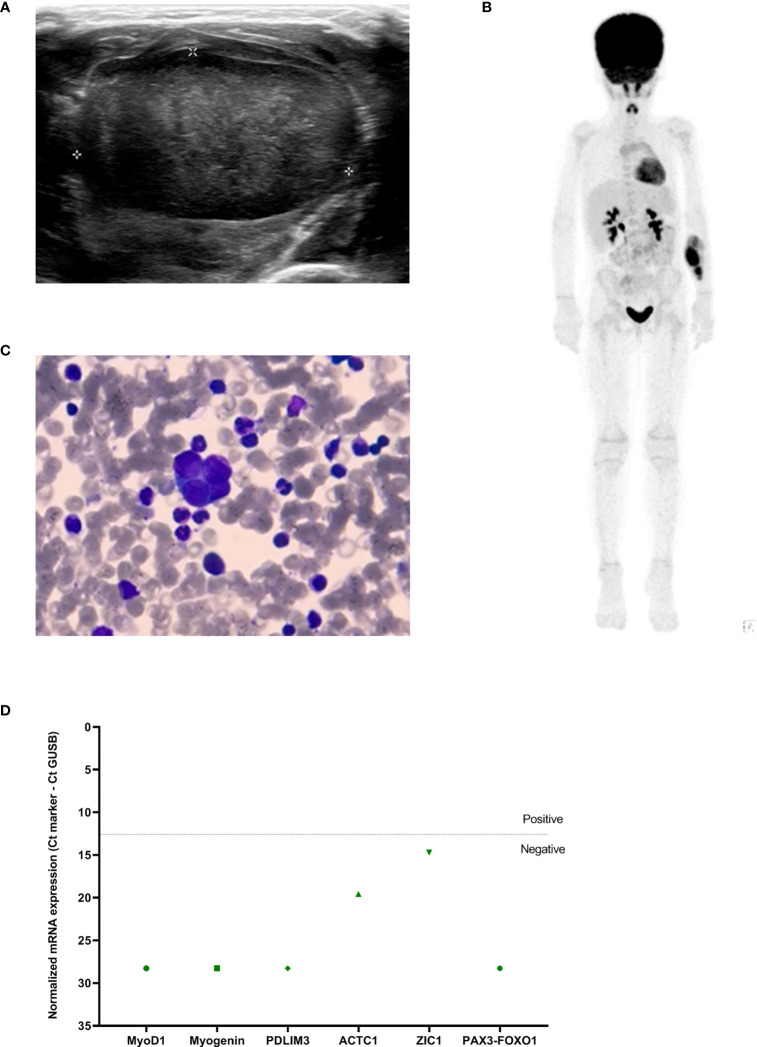
**(A)** Ultrasound and **(B)** [^18^F]FDG-PET at diagnosis showing the tumor in the left forearm, **(C)** bone marrow aspirate at diagnosis (63x enlarged) and **(D)** RT-qPCR rhabdomyosarcoma mRNA marker-panel results at diagnosis (Y-axis, normalized mRNA expression noted as Ct_marker_ - Ct_GUSB_; green BM infiltration). Dashed line indicates separation of positivity.

A 5-year-old girl presented with a painless swelling in the left forearm (**Table 1**). Imaging by [^18^F]FDG-PET showed a soft tissue tumor without metastases (**Figures 6A, B**). Tumor biopsy showed an embryonal rhabdomyosarcoma. MyoD1 staining (heterogeneously expressed in embryonal rhabdomyosarcoma) was considered ambiguous in the bone marrow aspirate (**Figure 6C**). A RT-qPCR using a rhabdomyosarcoma mRNA-panel (30) was then performed on the archived diagnostic BM sample. No tumor invasion was detected (**Figure 6D**), and the patient was diagnosed with non-metastatic rhabdomyosarcoma. This was later confirmed when the immunohistochemistry staining was reevaluated. The patient was treated according to a localized treatment protocol (RMS2005).

### Case 6

**Figure 7:**
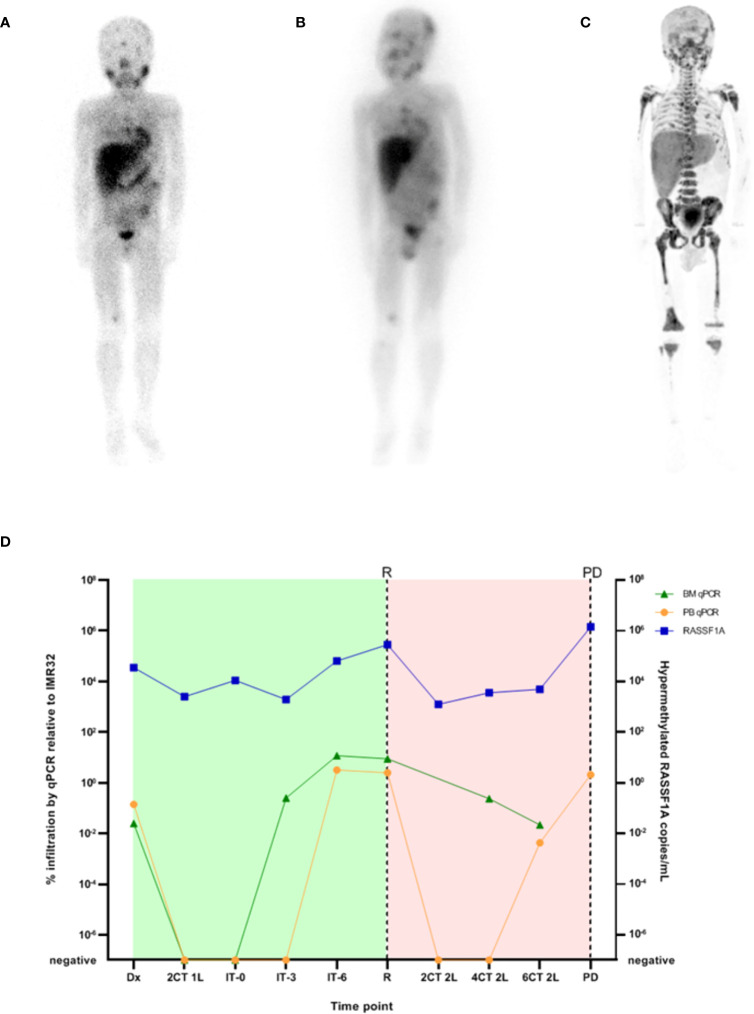
**(A)** [^123^I]mIBG scintigraphy before [^131^I]mIBG-treatment, **(B)** 3-day post [^131^I]mIBG-therapy scan **(C)** [^18^F]FDG-PET-scan after [^131^I]mIBG-treatment and **(D)** RT-qPCR neuroblastoma mRNA marker-panel results (left Y-axis, percentage infiltration as calculated by IMR32; green triangles BM infiltration, orange circles PB infiltration) and ddPCR *RASSF1A* methylation-assay (right Y-axis, copies hypermethylated *RASSF1A* per mL plasma, blue squares). Dx, diagnosis; nCT 1L, after n courses in first line therapy; nCT 2L, after n courses in second line therapy; IT-0, before GD-2 immunotherapy; IT-3, after 3 cycles of GD-2 immunotherapy; IT-6, after 6 cycles of GD-2 immunotherapy; R, relapse; PD, progressive disease. Green block indicates first line treatment, red block indicates treatment for relapsed disease.

A 6-year-old boy, treated for a high-risk neuroblastoma, suffered from a bone marrow recurrence two years after diagnosis (**Table 1**). During relapse treatment, [^123^I]mIBG imaging showed progressive disease for which he was treated with [^131^I]mIBG therapy (**Figure 7A**). After [^131^I]mIBG therapy (**Figure 7B**), he suffered from pancytopenia despite autologous hematopoietic stem cell reinfusion and severe hemorrhagic cystitis for which he received continuous bladder irrigation through a urinary catheter. When further progression of the disease was suspected, an [^18^F]FDG-PET scan was performed, as [^123^I]mIBG scans are less reliable post-[^131^I]mIBG therapy. This demonstrated diffuse bone marrow activation, reduced uptake in regions with known skeletal metastases and an enhanced liver uptake (**Figure 7C**). As a similar image can be caused by radionuclide therapy imaging and bone marrow activation, these results could not confirm our clinical suspicions. Urinary catecholamines could not be measured due to the continuous bladder irrigation. As an alternative, RT-qPCR was performed on a PB sample using our neuroblastoma mRNA marker-panel (28, 29), revealing exceedingly high expression of all markers. BM was not tested due to the patient’s clinical condition. In the same sample, a high percentage of *RASSF1A*-M was found in plasma using the *RASSF1A*-M ddPCR. On retrospective analysis, all sequentially collected liquid biopsies of this patient reflected the tumor burden and predicted his recurrence and progressive disease months before the actual clinical diagnosis. As shown in **Figure 7D**, *RASSF1A*-M stayed positive throughout and BM RT-qPCR became positive at immunotherapy, followed by the PB RT-qPCR. The liquid biopsy analyses, both the *RASSF1A*-M ddPCR and BM mRNA RT-qPCR, clearly indicated progressive disease. This resulted in initiating palliative therapy and the patient died a week after the analyses were performed.

## Discussion

Liquid biopsies are revolutionizing adult and pediatric oncology care. In this paper we highlight multiple potential uses and benefits of liquid biopsy, both at initial diagnosis by determining the tumor subtype or metastatic status, as well as for disease monitoring during treatment. Generally, the term liquid biopsy is used to refer to the analysis of fractions isolated from peripheral blood samples such as circulating cfDNA, cell-free RNA (cfRNA), or circulating tumor cells (CTCs). However, bone marrow, cerebrospinal fluid, urine and even saliva or pleural effusions may also be sampled. Novel components for analysis include extracellular vesicles, (tumor-educated) platelets, proteins and immune cells (22, 23). In **Table 2**, we highlight several existing publications on the techniques presented in this paper for the most common extra-cranial solid tumors in children. It must be noted that this is a non-comprehensive overview that does not include all existing literature and does not report on other techniques to detect MRD such as flow cytometry and cytology (55, 56).

**Table 2:** Liquid biopsy research.

Author	Tumor type(s)	Technique(s)	Body fluid(s)	Component
Diagnosis				
Van Paemel 2019 (23)	Neuroblastoma, renal tumors, rhabdomyosarcoma, osteosarcoma, Ewing sarcoma	cfRRBS	Plasma, CSF	cfDNA
Jiménez 2019 (4)	Renal tumors	WES	Plasma	
Peneder, Stütz 2021 (34)	Rhabdomyosarcoma, osteosarcoma, Ewing sarcoma	WGS, ddPCR	Plasma	
Shukla 2017 (30)	Ewing sarcoma	Capture-based NGS, ddPCR	Plasma	
Prognosis				
Stutterheim 2008 (15), 2011 (13)	Neuroblastoma	qPCR	BM	mRNA
Burchill 2001 (44)	Neuroblastoma	RT-qPCR	PB	
Van Wezel 2015 (16), 2016 (17)	Neuroblastoma	qPCR	PBSC, BM	
Viprey 2014 (45)	Neuroblastoma	RT-qPCR	BM, PB	
Yanez 2011 (46)	Neuroblastoma	RT-qPCR	BM, PB	
Lak 2021 (21)	Rhabdomyosarcoma	RT-qPCR	BM, PB	
Schleiermacher 2003 (47)	Ewing sarcoma	RT-PCR	BM, PB	
Chicard 2016 (3)	Neuroblastoma	OncoScan Array	Plasma	cfDNA
Van Roy 2017 (6)	Neuroblastoma	sWGS	Plasma	
Lodrini 2017 (24), 2022 (25)	Neuroblastoma	ddPCR	Plasma	
Van Wezel 2015 (18)	Neuroblastoma	qPCR	BM	
Gotoh 2005 (32)	Neuroblastoma	qPCR	PB	
Iehara 2019 (42)	Neuroblastoma	qPCR	PB	
Kojima 2013 (48)	Neuroblastoma	qPCR	Plasma	
Misawa 2009 (43)	Neuroblastoma	Methylation-specific PCR	PB	
Yagyu 2008 (40), 2011 (39)	Neuroblastoma	qPCR, microsatellite analysis	PB	
Stutterheim 2012 (14)	Neuroblastoma	qPCR	BM	
Combaret 2002 (31), 2009 (30), 2015 (29)	Neuroblastoma	qPCR, ddPCR	PB, plasma	
Van Paemel 2022 (5)	Neuroblastoma, rhabdomyosarcoma, Wilms tumor, osteosarcoma, Ewing sarcoma	sWGS	Plasma	
Klega 2018 (37)	Neuroblastoma, rhabdomyosarcoma, Wilms tumor, osteosarcoma	Ultra-low passage WGS	Plasma	
Charlton 2014 (22)	Wilms tumor	Bisulfite-sequencing	PB	
Madanat-Harjuoja 2022 (38)	Wilms tumor	NGS	PB, urine	
Treger 2018 (49)	Wilms tumor	ddPCR	PB, plasma, urine	
Shulman 2018 (36)	Osteosarcoma, Ewing sarcoma	NGS hybrid capture assay, ultra-low passage WGS	Plasma	
Shukla 2017 (30)	Ewing sarcoma	Capture-based NGS, ddPCR	Plasma	
Krumbholz 2021 (12)	Ewing sarcoma	ddPCR	Plasma	
Peneder, Stütz 2021 (34)	Rhabdomyosarcoma, osteosarcoma, Ewing sarcoma	WGS, ddPCR	Plasma	
Disease monitoring				
Van Wezel 2015 (16), 2016 (17)	Neuroblastoma	qPCR	PBSC, BM	mRNA
Marachelian 2017 (50)	Neuroblastoma	RT-qPCR	BM, PB	
Stutterheim 2008 (15), 2011 (13)	Neuroblastoma	qPCR	BM	
Van Zogchel 2021 (20)	Neuroblastoma	qPCR	BM, PB	
Lak 2021 (21)	Rhabdomyosarcoma	RT-qPCR	BM, PB	
Schleiermacher 2003 (47)	Ewing sarcoma	RT-PCR	BM, PB	
Chicard 2018 (7)	Neuroblastoma	WES	Plasma	cfDNA
Stutterheim 2012 (14)	Neuroblastoma	qPCR	BM	
Van Wezel 2015 (18)	Neuroblastoma	qPCR	BM	
Lodrini 2022 (25, 26)	Neuroblastoma	ddPCR	Plasma, BM, PB, CSF, urine	
Yagyu 2008 (40)	Neuroblastoma	qPCR	PB	
Van Zogchel 2020 (9), 2021 (19)	Neuroblastoma, renal tumors, rhabdomyosarcoma	qPCR, ddPCR	BM, PB, plasma	
Klega 2018 (37)	Neuroblastoma, rhabdomyosarcoma, Wilms tumor, osteosarcoma	Ultra-low passage WGS	Plasma	
Charlton 2014 (22)	Wilms tumor	Bisulfite-sequencing	PB	
Treger 2018 (49)	Wilms tumor	ddPCR	PB, plasma, urine	
Jiménez 2019 (4)	Renal tumors	WES	Plasma	
Eguchi-ishimae 2019 (51)	Rhabdomyosarcoma	qPCR	Plasma	
Barris 2018 (52)	Osteosarcoma	NGS	Plasma	
Shukla 2017 (30)	Ewing sarcoma	Capture-based NGS, ddPCR	Plasma	
Krumbholz 2016 (11), 2021 (12)	Ewing sarcoma	ddPCR	Plasma	
Hayashi 2016 (53)	Ewing sarcoma	ddPCR	Plasma	
Allegretti 2018 (54)	Ewing sarcoma	qPCR, ddPCR	Plasma	
Peneder, Stütz 2021 (34)	Rhabdomyosarcoma, osteosarcoma, Ewing sarcoma	WGS, ddPCR	Plasma	

Molecular profiling of tumor-derived cfDNA strongly correlates with tumor tissue (37, 38, 57). As ctDNA contains genetic alterations from the primary tumor in its entirety as well as its metastases, liquid biopsy can reduce sampling bias and increase diagnostic sensitivity (7, 22, 58–60).

Sample collection is mostly minimally invasive and can be repeated throughout treatment and follow-up, providing new opportunities to monitor disease burden and clonal evolution (6, 41, 61, 62). During follow-up, an increase in tumor-specific markers can signal a relapse (61).

Although the potential of liquid biopsy is clear, the development of specialized pediatric assays has been slow, challenged by the low incidence of pediatric tumors and their genetic heterogeneity. Pediatric tumors are inherently different from adult tumors. Histologically, embryonal and developmental cancers are more prevalent in children than in adults (63). Moreover, there is a marked difference in tumor-driving aberrations in pediatric cancers versus adult cancers. Pediatric cancers carry a low mutational burden and few recurrent hotspot mutations (single nucleotide variations). Instead, they predominantly show structural chromosomal variations (64, 65). As a result, assays validated for cfDNA detection in adults, which often target hotspot mutations such as *BRAF*, *EGFR*, or *KRAS*, do not translate to the pediatric population where such recurrent mutations are rare. Additionally, while sample volume is not typically a concern in adults, pediatric assays must be optimized to accommodate their smaller circulating volume. Together, these discrepancies underline the need to develop pediatric assays independently (23) and are the focus of most reviews on liquid biopsy approaches in pediatric solid tumors. Current studies optimizing the preanalytical workflow for liquid biopsies in pediatric patients have demonstrated its feasibility to perform these studies in small sample volumes at diagnosis, during therapy and at follow-up (66–69).

As highlighted in this paper, the analysis of mRNA and cfDNA has several potential applications for diagnosis, risk stratification and monitoring of therapy response (**Table 2**).

### Diagnosis

In cases 1, 2 and 3, we describe the use of cfDNA for diagnostic purposes. Tumor histology is known to correlate with the tumor’s DNA methylation pattern (70, 71). Clinically, methylation analysis is performed using an EPIC array, which is costly and requires a relatively large amount of DNA. In cases where tumor biopsy cannot be performed, such as with patient 2 and 3, methylation analysis of cfDNA could be used as a substitute diagnostic tool. cfRRBS on cfDNA is cost-effective and feasible even with small pediatric sample volumes (39). In cases 1 and 2, retrospective analysis of the diagnostic cfDNA samples resulted in a clear diagnosis. Particularly for patient 1, an earlier diagnosis of a second primary tumor might have resulted in a more patient-tailored treatment, although the rarity of the case precludes any certainty. With case 2, the uncertainty of the primary diagnosis is reflected in the result of the methylation analysis, which clearly shows that the *SMARCA4* loss detected in this patient correlates with a specific methylation pattern detected in cfDNA. However, unlike conventional tissue analysis, the cfRRBS methylation pattern also indicated neuroblastoma. Previous research has shown that loss of *SMARCA4* in neuroblastoma does occur and correlates with poor prognosis (72). In this case, we demonstrated that the methylation pattern of this tumor had a distinct signature which indicated both a rhabdoid tumor and a neuroblastoma. Although the outcome of cfRRBS did not directly impact clinical decision making in this case, the effect of the *SMARCA4* mutation on the methylation pattern indicates opportunities for further research on the diagnostic and prognostic value of this technique. Furthermore, case 2 illustrates how mRNA positivity of BM and PB for neuroblastoma-specific markers supported the diagnosis of metastatic neuroblastoma. Currently, several tumor-specific mRNA panels are being validated for clinical application in neuroblastoma and rhabdomyosarcoma tumors. For neuroblastoma, it has been shown repeatedly that tumor-derived mRNA can be sensitively detected in bone marrow and blood and that both correlate with clinical outcome (26, 44, 45, 73, 74). Newly developed MPX mRNA panels combining these markers decrease the required volume of blood or BM, the turnaround time and the (financial) resources required for the procedure (40).

### Prognosis

The prognostic use of cfDNA is shown in case 4, a patient in whom no amplification of *MYCN*, nor methylated *RASSF1A* was detected, and who therefore was treated according to a non-high-risk protocol. cfDNA-based detection of *MYCN* amplification has been described using qPCR (75, 76) and more recently ddPCR (42, 43). Hypermethylation of the tumor suppressor gene *RASSF1A* was shown to correlate with prognosis in neuroblastoma, rhabdomyosarcoma and Wilms tumors (32, 77–80). Our group has previously shown *RASSF1A*-M to correlate with outcome in neuroblastoma when it is detected in both the primary tumor and metastasized tumor cells in the BM (78). Of note, tumors with *MYCN* amplification had significantly higher *RASSF1A*-M levels than tumors without amplified *MYCN* (78). Detection of *RASSF1A*-M by ddPCR in plasma of patients with high-risk neuroblastoma clearly showed a significantly poorer event-free survival when hypermethylated *RASSF1A* exceeded 27,681 copies/mL at diagnosis (32). In rhabdomyosarcoma, detectable levels of *RASSF1A*-M in diagnostic plasma samples clearly correlate with poor outcome, especially in patients with metastatic disease (80). *RASSF1A*-M detection could therefore be complementary to the rhabdomyosarcoma RT-qPCR-panel (80).

In case 5, the rhabdomyosarcoma-specific mRNA panel was negative. A cohort study performed by our group showed that this panel is more sensitive than conventional diagnostic techniques to detect metastatic rhabdomyosarcoma (30). Its negativity was therefore sufficient to confirm that no BM infiltration had occurred. Case 5 demonstrates how panels such as these could assist to establish metastatic status and subsequent risk profile.

### Disease monitoring

In case 6, neuroblastoma-specific mRNA levels significantly increased prior to the first event and failed to decrease during second line therapy, which was in line with the clinical course of the disease. The extremely elevated mRNA-markers in PB confirmed progressive disease rather than toxic bone marrow depression. Furthermore, case 6 illustrates that *RASSF1A*-M detection enables sensitive monitoring of response to therapy (32, 41, 78). For this patient with poor outcome, *RASSF1A*-M analyzed by ddPPCR never became undetectable, but fluctuated between 10 (3)-10 (6) copies *RASSF1A*-M/mL plasma (**Figure 7D**). It should be noted that for disease monitoring, evidence indicates that combining cfDNA and mRNA analysis appears to increase sensitivity (41). A combination of ddPCR and RT-qPCR enables a more accurate quantitation of tumor markers (32).

## Conclusion and future prospects

Liquid biopsy shows great potential to assist with diagnosis, prognosis and disease monitoring in pediatric solid tumors. As liquid biopsies in pediatric oncology have not yet moved from bench to bedside, many techniques still await standardization. However, in a first step towards translation and clinical implementation, the techniques described in this case series are now being validated in international prospective trials. RT-qPCR and ddPCR will be validated for neuroblastoma in the ongoing SIOPEN HR-NBL2 trial. cfRRBS has been included in the ongoing SIOP RTSG-UMBRELLA trial for renal tumors. For rhabdomyosarcoma, the RNA panel and cfDNA analysis will be validated in the FaR-RMS trial. We recommend that liquid biopsies be used in conjunction with other conventional methods for the determination of tumor dissemination, prognostication and the monitoring of treatment response for pediatric patients with solid tumors. However, as illustrated by the case series in this paper, liquid biopsies may also be of crucial importance in cases where conventional techniques fall short.

## Data availability statement

The raw data supporting the conclusions of this article will be made available by the authors, without undue reservation.

## Ethics statement

Written informed consent was obtained from the minor(s)’ legal guardian/next of kin for the publication of any potentially identifiable images or data included in this article.

## Author contributions

NG and AB are shared first author. All authors contributed to the article and approved the submitted version.
