# Supplementary material for: Case series on clinical applications of liquid biopsy in pediatric solid tumors: towards improved diagnostics and disease monitoring
Source: Front Oncol. 2023 Aug 17;13:1209150. doi: 10.3389/fonc.2023.1209150 (PMC10473251; doi:10.3389/fonc.2023.1209150)
Supplement: Supplementary file 1 [file Table_1.docx]

**Supplemental data**

*Quantitative real-time PCR*

For the patients included in this report, tumor-derived mRNA in the cellular fraction of bone marrow (BM) aspirates and/or peripheral blood (PB) was detected. For neuroblastoma, a multiplex (MPX) adrenergic marker panel including *TH*, *CHRNA3* and *GAP43* and *DBH* for BM and PB and *PHOX2B* and the housekeeping gene glucuronidase beta (*GUSB*) as single markers was performed as previously described.^28,29^ Quantitative real-time PCR (RT-qPCR) was performed on the Viia7 (Applied Biosystems, Carlsbad, CA, USA), analysis was performed using QuantStudio software version 1.6 (Applied Biosystems).^40,41^ For rhabdomyosarcoma, the MPX panel included *MYOD1*, *MYOG*, *PDLIM3*, *ACTC1*, *ZIC1* and *PAX3/7-FOXO1* in BM and *SNAI2*, *CDH11*, *THEM47* and *MEGF10* in PB and performed as previously described.^41^ All RT-qPCR reactions were performed in triplicate (except *GUSB*, which was performed in duplicate) and mean values were used for analysis. Detection of neuroblastoma-specific mRNAs by RT-qPCR reliably detects with a sensitivity of 1 in 10^6^ normal nucleated bone marrow cells.^29^ If more than one BM sample of a patient was available at a certain time point (in case of bilateral sampling), samples were tested separately.

Data analysis of the RT-qPCR results was performed as previously described.^29,40,41^ Expression was normalized to *GUSB* expression using the following equation: [normalized threshold cycle (ΔCt) = Ct_marker_ – Ct*_GUSB_*]. Positivity of samples was scored according to earlier published thresholds.^29,40,41^

*Droplet Digital PCR*

In this report, detection of methylated *RASSF1A* (*RASSF1A*-M) in ctDNA was performed using the method described by Van Zogchel *et al*.^32,42^ This method subjects every sample to two different droplet digital PCR (ddPCR) reactions after cfDNA extraction, with and without the addition of methylation-sensitive restriction enzymes (MRSE), all remaining conditions are identical. ACTB-1 primer-probe set was added to control for cfDNA input, as this amplicon is unaffected by the MRSE. ACTB-2 primer-probe set was added to control for MRSE performance since this amplicon is digested by the enzymes. To avoid false-positive samples, threshold is set at four positive droplets per duplicate. If a sample was scored positive, the percentage of hypermethylated *RASSF1A* was calculated as (*RASSF1A*/*ACTB*^with MRSE^)/(*RASSF1A*/*ACTB*^without MRSE^) x 100%.

Amplified *MYCN* is a prognostic high-risk feature in neuroblastoma.^33^ The *MYCN* copy number status in patients with neuroblastoma can be detected by ddPCR using cfDNA in blood plasma. For the patients in this report, a *MYCN* primer-probe set and an *NAGK* (normal diploid reference gene) primer-probe set were added in a duplex PCR reaction. *MYCN* copy number was then determined by calculating the ratio of *MYCN* to the reference gene.^43,71^

*Reduced representation bisulphite sequencing of cell-free DNA (cfRRBS)*

Methylation analysis of cfDNA using cell-free reduced representation bisulphite sequencing (cfRRBS) was performed as previously described.^39^ The diagnosis is established based on the highest estimated tumor fraction using reference-based deconvolution as described by Moss *et al.* (<https://github.com/nloyfer/meth_atlas>).^45^
